# Comparative Efficacy and Safety of Antihypertensive Agents for Adult Diabetic Patients with Microalbuminuric Kidney Disease: A Network Meta-Analysis

**DOI:** 10.1371/journal.pone.0168582

**Published:** 2017-01-03

**Authors:** Rongzhong Huang, Yuxing Feng, Ying Wang, Xiaoxia Qin, Narayan Dhruvaraj Melgiri, Yang Sun, Xingsheng Li

**Affiliations:** 1 Department of Rehabilitation Medicine, the Second Affiliated Hospital of Chongqing Medical University, Chongqing, China; 2 Department of Neurology, the Ninth People’s Hospital of Chongqing, Chongqing, China; 3 Impactys Foundation for Biomedical Research, San Diego, CA, United States of America; 4 Institute of Ultrasound Imaging, the Second Affiliated Hospital of Chongqing Medical University, Chongqing, China; 5 Department of Gerontology, the Second Affiliated Hospital of Chongqing Medical University, Chongqing, China; University of Glasgow, UNITED KINGDOM

## Abstract

**Background:**

Antihypertensive treatment mitigates the progression of chronic kidney disease. Here, we comparatively assessed the effects of antihypertensive agents in normotensive and hypertensive diabetic patients with microalbuminuric kidney disease.

**Methods:**

MEDLINE, EMBASE, and the Cochrane Central Register of Controlled Trials were systematically searched for randomized controlled trials (RCTs) comparing oral antihypertensive agents in adult diabetic patients with microalbuminuria. The primary efficacy outcome was reduction in albuminuria, and the primary safety outcomes were dry cough, presyncope, and edema. Random-effects pairwise and Bayesian network meta-analyses were performed to produce outcome estimates for all RCTs, only hypertensive RCTs, or only normotensive RCTs. Surface under the cumulative ranking (SUCRA) probability rankings were calculated for all outcomes. Sensitivity analyses on type 2 diabetes status, age, or follow-up duration were also performed.

**Results:**

A total of 38 RCTs were included in the meta-analyses. The angiotensin-converting enzyme inhibitor-calcium channel blocker (ACEI-CCB) combination therapy of captopril+diltiazem was most efficacious in reducing albuminuria irrespective of blood pressure status. However, the ACEI-angiotensin receptor blocker (ACEI-ARB) combination therapy of trandolapril+candesartan was the most efficacious in reducing albuminuria for normotensive patients, while the ACEI-CCB combination therapy of fosinopril+amlodipine was the most efficacious in reducing albuminuria for hypertensive patients. The foregoing combination therapies displayed inferior safety profiles relative to ACEI monotherapy with respect to dry cough, presyncope, and edema. With respect to type 2 diabetic patients with microalbuminuria, the Chinese herbal medicine Tangshen formula followed by the ACEI ramipril were the most efficacious in reducing albuminuria.

**Conclusions:**

Trandolapril+candesartan appears to be the most efficacious intervention for reducing albuminuria for normotensive patients, while fosinopril+amlodipine appears to be the most efficacious intervention for reducing albuminuria for hypertensive patients. For practitioners opting for monotherapy, our SUCRA analysis supports the use of trandolapril and fosinopril in normotensive and hypertensive adult diabetic patients with microalbuminuria, respectively.

## Introduction

Diabetes mellitus affects ~4% of the global adult population with an estimated 382 million affected individuals in 2013 expected to increase to an estimated 592 million affected individuals by the year 2035 [1]. Diabetes is the primary cause of end-stage kidney disease (ESRD), contributing to 40–50% of chronic dialysis patients [2]. As the increasing number of diabetic individuals is projected to have a serious impact on dialysis service and kidney transplant needs, the development of cost-effective therapeutic strategies for individuals with diabetic kidney disease is a crucial public health concern [3].

As it is well-established that blood pressure (BP) control is critical to slowing the decline in the glomerular filtration rate (GFR) [4], antihypertensive treatment has been credited with mitigating the progression of chronic kidney disease (CKD) to ESRD [5]. Specifically, clinical guidelines recommend a 130/80 mm Hg BP target as well as the use of angiotensin-converting enzyme inhibitors (ACEIs) and angiotensin receptor blockers (ARBs) as first-line agents for BP control in CKD patients [5]. Indeed, a recent network meta-analysis consisting of 157 trials by Palmer et al. that comparatively assessed the efficacy and safety of BP-lowering agents in adult diabetic CKD patients found that ESRD risk was significantly reduced after combined treatment with an ACEI and an ARB [6].

However, Palmer et al.’s network meta-analysis specifically noted a limitation to their study: their ESRD outcomes were largely restricted to adult diabetic patients with macroalbuminuric kidney disease [6]. Thus, their results cannot be generalized to adult diabetic patients with microalbuminuric kidney disease. Moreover, although Palmer et al. did conduct a sensitivity analysis restricted to adult diabetic patients with microalbuminuria [6], their group did not comparatively examine normotensive versus hypertensive patients within this cohort. The examination of antihypertensive agents in reducing albuminuria in adult diabetic patients with microalbuminuria is of particular clinical importance for these patients, as (i) microalbuminuria in adult diabetic patients has been identified as a risk factor for adverse cardiovascular events, and (ii) a failure to control increasing albuminuria in these patients (after controlling for other risk factors) heightens the risk of inferior renal outcomes [7]. Moreover, the question is of importance to healthcare practitioners, as optimal long-term management of adult diabetic patients with microalbuminuria requires evidence-based recommendations on the efficacy and safety of various antihypertensive agents that are specifically tailored to this patient population [8].

Therefore, the aim of this network meta-analysis will be to assess the comparative effects of antihypertensive agents in reducing albuminuria in adult diabetic patients with microalbuminuric kidney disease. Moreover, we will specifically examine the comparative effects of antihypertensive agents in reducing albuminuria in normotensive versus hypertensive patients within this microalbuminuric cohort.

## Methods

### Study Design

This network meta-analysis was conducted according to the Preferred Reporting Items of Systematic Reviews and Meta-Analyses (PRISMA) statement [9]. This network meta-analysis–which integrates direct treatment comparisons within trials as well as indirect treatment comparisons compared against a common comparator across separate randomized controlled trials (RCTs) [10]–was performed using a frequentist analytical approach [11].

### Search Strategy

MEDLINE, EMBASE, and the Cochrane Central Register of Controlled Trials were comprehensively searched up to October 2015 for relevant RCTs using the following search terms: (diabetes or diabetic) AND (antihypertensive OR “blood pressure-lower*” OR “blood pressure-reduc*”) AND microalbuminur* AND random* AND control*. An English language restriction was imposed on all searches.

### Inclusion and Exclusion Criteria

Two investigators (Rongzhong Huang and Yang Sun) were responsible for independently selecting the studies, with any conflicts resolved through discussion. The inclusion and exclusion criteria were based on Palmer et al.’s previously published criteria with minor modifications [6].

The inclusion criteria for this network meta-analysis were as follows: (i) parallel-group RCT design, (ii) minimal eight-week follow-up period, (iii) adult participants (individuals aged 18 years or older) with diabetes and microalbuminuric kidney disease (i.e., evidenced by a urinary albumin excretion rate (UAER) > 30 mg/day), (iv) comparing an oral antihypertensive agent (alone or in combination) (e.g., ACEI, ARB, calcium-channel blocker (CCB), β-blocker, α-blocker, diuretic, renin inhibitor, aldosterone antagonist, or endothelin inhibitor) against a second antihypertensive agent or combination therapy, placebo, or control, (v) specifically analyzing and reporting on either a hypertensive, normotensive, or a mixed population and (vi) measuring UAER as an outcome. Both fixed-dose and flexible-dose RCTs with dose titration were included. We also included RCTs with a general population of adult diabetes participants when the data for those participants who did have microalbuminuric kidney disease could be extracted separately.

The exclusion criteria for this network meta-analysis were as follows: (i) studies in children and adolescents (defined as individuals aged under 18 years), (ii) studies including patients with microalbuminuric kidney disease secondary to causes other than diabetes (if specified), (iii) studies including patients with an active kidney transplant, undergoing kidney transplantation, or undergoing dialysis (if specified), (iv) studies failing to report the number of patients, (v) studies failing to report the mean value of the primary efficacy outcome, or (vi) studies failing to report the data necessary to estimate the standard deviation (SD) of the primary efficacy outcome.

### Outcomes

As albumin excretion has been shown to increase in diabetic patients prior to the development of diabetic nephropathy [12], the primary efficacy outcome was defined as reduction in albuminuria as expressed by the UAER. Specifically, for each included study, the mean percentage (%) reduction in UAER for each normotensive cohort and each hypertensive cohort were calculated in order to enable normotensive versus hypertensive comparisons. The primary safety outcomes were dry cough, presyncope, and peripheral edema.

### Data Extraction

Two investigators (Rongzhong Huang and Yang Sun) were responsible for independently extracting the data into a spreadsheet according to a predefined protocol, with any conflicts resolved through discussion. The following parameters were extracted from each included RCT: first author’s name, year of publication, study location, mean age of participants, type of diabetes among participants, definition of microalbuminuria, BP categories of participants, intervention(s) prescribed (with dosage levels), and follow-up period (in months), the primary efficacy outcome of UAER (standardized to μg/min), and the primary safety outcomes of dry cough, presyncope, and peripheral edema.

### Risk of Bias Assessment

Risk of bias was assessed using the approach recommended by the Cochrane reviews. The following bias domains were independently assessed by two investigators (Rongzhong Huang and Yang Sun): random sequence generation, allocation concealment, blinding of investigators and/or participants, blinding of outcome assessment, degree of incompleteness of outcome data, and selective reporting of study outcomes. Each bias item was scored as ‘low risk’, ‘unclear risk’, or ‘high risk’.

### Statistical Analysis

Standard pairwise and network meta-analyses were performed to estimate the primary efficacy outcome. Prior to performing the meta-analyses, Stata version 13 was first used to estimate the SDs for studies that failed to report SDs using (i) interquartile ranges, (ii) the 95% confidence intervals (CIs) of the primary efficacy outcome, and (iii) the two-sided *p*-value corresponding to the *t*-test between the two treatment modalities.

Then, using R. version 3.2.2 (metaphor package and R routines), a random-effects model was used to perform the standard pairwise meta-analysis. Heterogeneity was assessed with the I^2^ metric. For the primary data analysis, we used the intention-to-treat principle. In studies that only reported a per-protocol analysis, we conservatively assumed that all dropouts were treatment failures for inclusion in the intention-to-treat analysis; this procedure guarded against favoring active drugs that could be the more harmful [13, 14]. The estimates of the primary efficacy outcome were presented as standardized mean differences (SMDs) with 95% CIs.

Next, a random-effects network meta-analysis was performed in ADDIS version 1.16.6 –a software package employing Bayesian Markov chain Monte Carlo methods—assuming a common heterogeneity variable (tau [τ]) for all comparisons. τ is the estimated SD of the underlying effects of treatment across the studies in a meta-analysis. RCTs with drugs that could not be connected to the network were excluded from the network meta-analysis and only drugs directly or indirectly connected to placebo were included in the downstream ranking analysis. Consistency of the RCTs included in the network was assessed through applying inconsistency and node-splitting models. The consistency results were considered insignificant when 95% CIs of inconsistency factors included zero or when the *p*-value was greater than 0.05 for the comparison between direct and indirect effects in the node splitting analysis. The estimates of the primary efficacy outcome were calculated as SMDs with 95% CIs, while the network meta-analyses estimates of the primary safety outcomes were calculated as odds ratios.

To rank the treatments, we calculated surface under the cumulative ranking (SUCRA) probabilities, which express as a percentage the efficacy or safety of each intervention relative to a hypothetical ideal intervention. Thus, a large SUCRA score indicates a more effective or safer intervention. Rankings were calculated for the primary efficacy outcome and the three primary safety outcomes.

In order to assess the effects of hypertensive status, the foregoing meta-analyses were also re-performed by pooling only hypertensive studies and pooling only normotensive studies. In addition, we performed three separate sensitivity analyses on type 2 diabetes status, age, or follow-up duration by measuring the effects of including only trials with type 2 diabetes participants, trials with participants aged 40 and older, or trials with follow-up durations of 12 months and longer, respectively.

## Results

**Fig 1:**
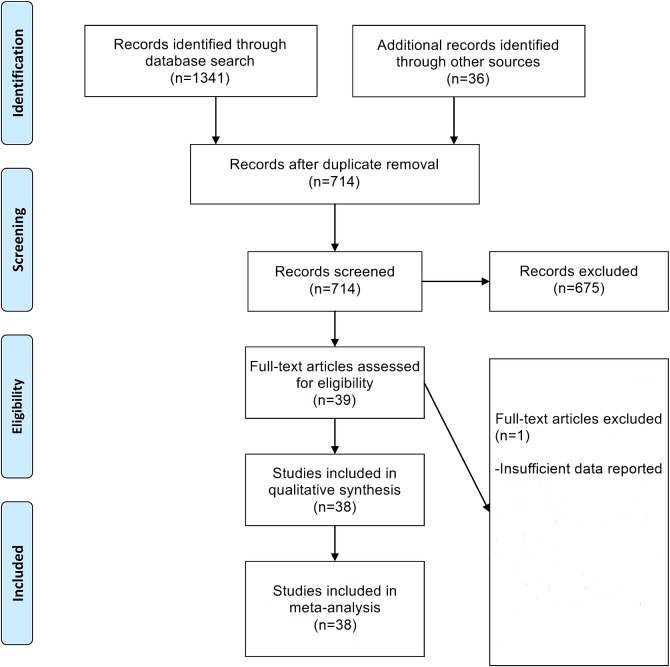
PRISMA Flowchart of Study Selection Process.

The PRISMA flowchart detailing the study selection process is provided in Fig 1. From an initial set of 714 non-duplicate records, a total of 38 RCTs were finally included in the meta-analysis. The characteristics of these included RCTs are detailed in Table 1. The risk of bias assessment for these included RCTs are detailed in Table 2.

**Table 1:** Characteristics of Included RCTs.

Study	Location	N	Mean age (range)	Diabetestype	Microalbuminuria definition	BP categories	Intervention(s)	Follow-up (months)
ABCD-2V (Estacio) 2006 [28]	USA	129	56.1 (40–81)	2	UAER 20–200 μg/min	Normotensive	Valsartan 80 mg/d, then valsartan 160 mg/d then HCTZ 12.5 mg/d, 25 mg/d, then metoprolol 50 mg/d, 100 mg twice a day; placebo	22.8
Atmaca 2006 [29]	Turkey	26	55.1 (36.7–73.5)*	2	UAER 30–300 mg/d	Normotensive	Lisinopril 10 mg/d, losartan 50 mg/d, lisinopril 10 mg/d + losartan 50 mg/d	12
Bojestig 2001 [30]	Sweden	55	39.6 (20.3–58.9)*	1	Urine albumin to creatinine ratio 2.5–25 mg/mmol	Normotensive	Ramipril 1.25 mg/d, ramipril 5 mg/d, placebo	48
CALM (Mogensen) 2000 [31]	Multi-national	199	60.0 (30–75)	2	Urine albumin to creatinine ratio 2.5–25 mg/mmol	Hypertensive	Lisinopril 20 mg/d, candesartan 16 mg/d, lisinopril 20 mg/d + candesartan 16 mg/d	3
ESTIMATE-A (Kojima) 2013 [32]	Japan	40	68.1 (20–75)	2	UAE> 30 mg/g creatinine and serum creatinine <1.5 mg/dl for men and <1.2 mg/dl for women	Hypertensive	Telmisartan 40–80 mg/d + TCMZ 1 mg/d, telmisartan 40–80 mg/d + TCMZ 1 mg/d + amlodipine 5 mg/d	6
EUCLID (Chaturvedi) 1997 [33]	UK	530	33.0 (20–59)	1	UAER 20–200 μg/min	Hypertensive	Lisinopril 10–20 mg, placebo	24
Fogari 1997a [34]	Italy	50	53.9 (51.6–56.1)*	2	UAER 30–300 mg/d;serum creatinine<1.4 mg/dl	Hypertensive	Amlodipine 10 mg/d, enalapril 20 mg/d	12
Fogari 1997b [34]	Italy	45	57.1 (54.7–59.5)*	2	UAER 30–300 mg/d;serum creatinine> = 1.3 mg/dl	Hypertensive	Benazepril 10 mg/d, benazepril 10 mg/d + amlodipine 5 mg/d	6
Fogari 2000 [35]	Italy	254	68.3 (60–75)	2	UAER 30–300 mg/d;serum creatinine<1.3 mg/dl	Hypertensive	Amlodipine 5–10 mg/d, fosinopril 10–20 mg/d, placebo	24
Fogari 2002 [36]	Italy	453	62.5 (44.4–80.7)*	2	UAER 30–300 mg/d;serum creatinine<1.5 mg/dl	Hypertensive	Fosinopril 10–30 mg/d, amlodipine 5–15 mg/d, fosinopril 10–30 mg/d + amlodipine 5–15 mg/d	48
Fogari 2005 [37]	Italy	121	60.3 (47.2–73.3)*	2	UAER 30–300 mg/d;serum creatinine<1.4 mg/dl	Hypertensive	Manidipine 10 mg/d, lisinopril 10 mg/d	24
Fogari 2007 [38]	Italy	174	55.7 (40–65)	2	UAER 30–300 mg/d	Hypertensive	Candesartan 16 mg/d + manidipine 10–20 mg/d, candesartan 16 mg/d + HCTZ 12.5–25 mg/d	6
Fogari 2012 [39]	Italy	109	65.0 (30–75)	2	UAER 30–300 mg/d	Hypertensive	Valsartan 160 mg/d + amlodipine 5 mg/d + canrenone 25–50 mg/d, valsartan 160 mg/d + amlodipine 5 mg/d + HCTZ 12.5–25 mg/d	6
Fogari 2013 [40]	Italy	176	60.8 (25–75)	2	UAER 200–300 mg/d	Hypertensive	Imidapril 10–20 mg/d, ramipril 5–10 mg/d	6
JAPAN-IDDM (Katayama) 2002 [41]	Japan	79	30.9 (20–50)	1	UAER>30 mg/d	Hypertensive	Captopril 37.5 mg/d, imidapril 5 mg/d, placebo	17.8
Jerums 2001 [42]	Australia	33	30.8 (16–65)	1	UAER 20–200 μg/min;serum creatinine<200 μmol/l	Normotensive	Perindopril 2–8 mg/d, nifedipine 20–80 mg/d, placebo	67.2
Josefsberg 1995 [43]	Canada	21	53.0 (37–68)	2	UAER 20–200 μg/min	Hypertensive	Nitrendipine 10–40 mg/d, enalapril 5–20 mg/d	7.5
Kohlmann 2009 [44]	Multi-national	110	63.3 (45–81)	2	Urine albumin to creatinine ratio 2.5–25 mg/mmol for men and 3.5–25 mg/mmol for women	Hypertensive	Manidipine 10 mg/d + delapril 30 mg/d, losartan 50 mg/d + HCTZ 12.5 mg/d	12
Lacourciere 2000 [45]	Canada	103	58.5 (38.8–78.2)*	2	UAER 20–350 μg/min;serum creatinine<1.7 mg/dl	Hypertensive	Losartan 50 mg/d, enalapril 5–10 mg/d	12
Li 2015 [46]	China	98	58.6 (38.8–78.3)*	2	UAER 20–200 μg/min, GFR 50–120 ml/min, serum creatinine 50–100 μmol/l	Mixed	Tangshen formula, placebo	6
MARVAL (Viberti) 2002 [47]	UK	368	58.0 (35–75)	2	UAER 20–200 μg/min;normal serum creatinine	Mixed	Valsartan 80 mg/d, amlodipine 5 mg/d	6
Muirhead 1999 [48]	Canada	122	56.0 (35.9–76.2)*	2	UAER 20–300 μg/min;GFR > = 60 ml/min	Mixed	Valsartan 80 mg/d, valsartan 160 mg/d, captopril 75 mg/d, placebo	12
Melbourne Diabetic Nephropathy Study Group (Doyle) 1991 [49]	Australia	43	50.0 (18–66)	1 and 2	UAER 20–200 μg/min;serum creatinine<2.3 mg/dl	Separate normotensive and hypertensive cohorts	Perindopril 2–8 mg/d, nifedipine 10–40 mg twice daily	12
Nakamura 2002 [50]	Japan	60	56.5 (37.6–75.4)*	2	UAER 20–200 μg/min	Normotensive	Trandolapril 2 mg/d, candesartan 8 mg/d, trandolapril 2 mg/d + candesartan 8 mg/d, placebo	18
Perez-Maraver 2005 [51]	Spain	36	60.4 (45.6–75.2)*	2	UAER 30–300 μg/min	Hypertensive	Captopril 25 mg/12 h-50 mg/8 h, captopril 25 mg/12 h-50 mg/8 h + diltiazem 120 mg/d	24
Poulsen 2001 [52]	Denmark	21	32.4 (18.0–52.2)*	1	UAER 20–70 μg/min	Normotensive	Lisinopril 20 mg/d, placebo	24
PREMIER (Mogensen) 2003 [53]	Multi-national	481	59.6 (40–75)	2	UAER 20–500 μg/min, serum creatinine<1.6 mg/dl	Hypertensive	Perindopril 2–8 mg/d + 0.625–2.5 mg/d indapamide, enalapril 10–40 mg/d	12
Sano 1994 [54]	Japan	48	63.5 (50–76)	2	UAER 20–500 μg/min, serum creatinine<140 μmol/l	Normotensive	Enalapril 5 mg/d, placebo	48
Sato 2003 [55]	Japan	50	63.3 (43.1–83.6)*	1 and 2	UAE 30–300 mg/g creatinine,GFR< = 60 ml/min	Separate hypertensive and normotensive cohorts	ACEI (trandolapril 1.5 mg/d or enalapril 7.5 mg/d), candesartan 7.1 mg/d	11
Schnack 1994 [56]	Austria	15	37.9 (29.9–45.9)*	1	UAER 30–300 mg/d;serum creatinine<1.4 mg/dl	Normotensive	Nifedipine 30 mg/d, placebo	12
Sengul 2006 [57]	Turkey	145	57.2 (40–65)	2	UAER 30–300 mg/d;creatinine< = 1.7 mg/dl	Hypertensive	Lisinopril 20 mg/d, telmisartan 80 mg/d, lisinopril 20 mg/d + telmisartan 80 mg/d (following lisinopril monotherapy), lisinopril 20 mg/d + telmisartan 80 mg/d (following telmisartan monotherapy)	7
Shigihara 2000 [58]	Japan	30	62.9 (56.0–69.7)*	2	Microalbuminuria	Hypertensive	ACEI (enalapril 7.0 mg/d, trandolapril 1.6 mg/d, or imidapril 9.2 mg/d), ACEI (enalapril 5.3 mg/d, trandolapril 1.6 mg/d, or imidapril 5 mg/d) + amlodipine 7.0 mg/d	3
Takebayashi 2006 [59]	Japan	37	N/R	2	UAE>30 mg/g creatinine	Mixed	Spironolactone 50 mg/d, amlodipine 2.5 mg/d	3
Tan 2002 [60]	China	80	54.5 (35.6–73.4)*	2	UAER 20–200 μg/min	Mixed	Losartan 50 mg/d, placebo	6
Tutuncu 2001 [61]	Turkey	34	55.6 (38.7–72.5)*	2	UAER 30–300 mg/d	Normotensive	Enalapril 5 mg/d, losartan 50 mg/d, enalapril 5 mg/d + losartan 50 mg/d	12
Weil 2013 [62]	USA	78	42.1 (22.3–61.9)*	2	ACR 30–300 mg/g;serum creatinine <1.4 mg/dl	Mixed	Losartan 50–100 mg/d, placebo	70.8
Viberti 1994 [63]	Multi-national	92	31.5 (18–54)	1	UAER 20–200 μg/min;serum creatinine<150 μmol/l	Normotensive	Captopril 100 mg/d, placebo	24
Zandbergen 2003 [64]	Netherlands	147	57.7 (34.4–81.0)*	2	UAER 20–200 μg/min; serum creatinine< = 1.7 mg/dl	Normotensive	Losartan 50–100 mg/d, placebo	2.5

*Age range estimated from the reported standard deviations (SDs) [65].

Abbreviations: UAE, urine albumin excretion; UAER, urine albumin excretion rate; ACR, albumin-to-creatinine ratio; GFR, glomerular filtration rate; ACEI, angiotensin-converting enzyme inhibitor; HCTZ, hydrochlorothiazide; TCMZ, trichlormethiazide

**Table 2:** Risk of Bias Assessment.

Study	Random sequence generation	Allocation concealment	Blinding of personnel and participants	Blinding of outcome assessment	Incomplete outcome data	Selective reporting	Other bias	Total score
ABCD-2V (Estacio) 2006 [28]	L	L	L	L	L	L	L	7
Atmaca 2006 [29]	L	U	U	U	L	L	H	3
Bojestig 2001 [30]	L	L	L	L	L	L	H	6
CALM (Mogensen) 2000 [31]	L	L	L	L	L	L	L	7
ESTIMATE-A (Kojima) 2013 [32]	L	U	U	U	L	L	h	3
EUCLID (Chaturvedi) 1997 [33]	L	U	l	U	L	L	L	5
Fogari 1997a [34]	L	U	U	U	L	L	H	3
Fogari 1997b [34]	L	U	U	U	L	L	H	3
Fogari 2000 [35]	L	L	L	L	L	L	L	7
Fogari 2002 [36]	L	U	U	U	L	L	L	4
Fogari 2005 [37]	L	U	U	U	L	L	H	3
Fogari 2007 [38]	L	L	L	L	L	L	L	7
Fogari 2012 [39]	L	L	L	L	L	L	L	7
Fogari 2013 [40]	L	L	L	L	L	L	L	7
JAPAN-IDDM (Katayama) 2002 [41]	L	U	U	U	L	L	H	3
Jerums 2001 [42]	L	L	L	L	L	L	H	6
Josefsberg 1995 [43]	L	U	U	U	L	L	H	3
Kohlmann 2009 [44]	L	L	L	L	L	L	L	7
Lacourciere 2000 [45]	L	L	L	L	L	L	H	6
Li 2015 [46]	L	L	L	L	L	L	L	7
MARVAL (Viberti) 2002 [47]	L	L	L	L	L	L	L	7
Muirhead 1999 [48]	L	U	l	U	L	L	H	4
Melbourne Diabetic Nephropathy Study Group (Doyle) 1991 [49]	L	L	L	L	L	L	H	6
Nakamura 2002 [50]	L	U	U	U	L	L	H	3
Perez-Maraver 2005 [51]	L	H	H	H	L	L	H	3
Poulsen 2001 [52]	L	L	L	L	L	L	H	6
PREMIER (Mogensen) 2003 [53]	L	U	U	U	L	L	H	3
Sano 1994 [54]	L	U	U	U	L	L	H	3
Sato 2003 [55]	U	U	U	l	L	L	H	3
Schnack 1994 [56]	L	L	L	L	L	L	H	6
Sengul 2006 [57]	L	U	U	U	L	L	L	4
Shigihara 2000 [58]	L	U	U	U	L	L	H	3
Takebayashi 2006 [59]	L	U	U	U	L	L	H	3
Tan 2002 [60]	L	L	L	L	L	L	L	7
Tutuncu 2001 [61]	L	U	U	U	L	L	H	3
Weil 2013 [62]	L	L	L	L	L	L	H	6
Viberti 1994 [63]	L	L	L	L	L	L	H	6
Zandbergen 2003 [64]	L	L	L	L	L	L	L	7

Abbreviations: L, low risk of bias; U, unclear risk of bias; H, high risk of bias

**Fig 2:**
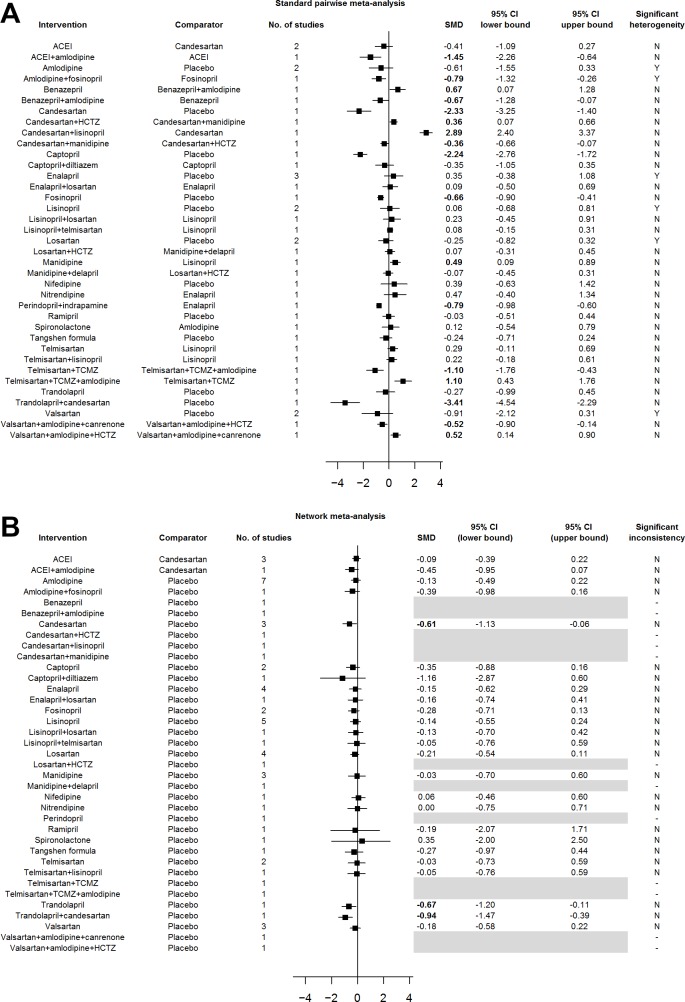
Standard Pairwise and Network Meta-Analyses of All RCTs. Results from the (A) standard pairwise meta-analysis and (B) network meta-analysis of all RCTs. Standardized mean differences (SMDs) and 95% confidence intervals (CIs) are presented with statistically significant SMDs denoted in bold font. Greyed-out areas represent comparisons that could not be connected into the network. Abbreviations: ACEI, ACE inhibitor (random selection of enalapril, trandolapril, or imidapril); HCTZ, hydrochlorothiazide; TCMZ, trichlormethiazide; SMD, standardized mean difference; 95% CI, 95% confidence interval.

First, standard pairwise meta-analysis (Fig 2A) and network meta-analysis (Fig 2B) were performed on all included RCTs. The network map diagramming the direct comparisons for this overall network meta-analysis is provided in S1 Fig. SUCRA analysis for the primary efficacy outcome of UAER reduction revealed that captopril+diltiazem was the most efficacious intervention, followed by trandolapril+candesartan, followed by trandolapril (Table 3). SUCRA analysis for the primary safety outcomes revealed that: (i) captopril, followed by captopril+diltiazem, followed by ramipril were the safest interventions for dry cough; (ii) ACEI (random selection of enalapril, trandolapril, or imidapril), followed by lisinopril+candesartan, and followed by lisinopril were the safest interventions for presynope, and (iii) lisinopril, followed by candesartan, followed by ACEI (random selection of enalapril, trandolapril, or imidapril) were the safest interventions for peripheral edema (Table 3).

**Table 3:** SUCRA-Based Primary Efficacy and Safety Outcome Rankings from All RCTs.

		Primary safety outcome rankings		
Intervention	Primary efficacy	Dry cough	Presyncope	Peripheral edema
	outcome ranking*	ranking*	ranking*	ranking*
ACEI	-	-	1	4
ACEI+amlodipine	-	-	-	-
Amlodipine	13	-	-	-
Amlodipine+fosinopril	5	-	-	-
Benazepril	-	-	-	-
Benazepril+amlodipine	-	-	-	-
Candesartan	4	-	5	3
Candesartan+HCTZ	-	-	-	-
Candesartan+lisinopril	-	-	3	5
Candesartan+manidipine	-	-	-	-
Captopril	6	1	-	-
Captopril+diltiazem	1	2	-	-
Enalapril	12	-	-	-
Enalapril+losartan	10	-	-	-
Fosinopril	8	-	-	-
Lisinopril	14	-	4	2
Lisinopril+losartan	15	-	-	-
Lisinopril+telmisartan^†^	18	-	-	-
Losartan	9	-	6	-
Losartan+HCTZ	-	-	-	-
Manidipine	19	-	-	6
Manidipine+delapril	-	-	-	-
Nifedipine	22	-	-	-
Nitrendipine	21	-	-	-
Perindopril	-	-	-	-
Perindopril+indapamide	-	-	-	-
Ramipril	23	4	-	-
Spironolactone	24	-	-	-
Tangshen formula	7	-	-	-
Telmisartan	20	-	-	-
Telmisartan+lisinopril^†^	17	-	-	-
Telmisartan+TCMZ	-	-	-	-
Telmisartan+TCMZ+amlodipine	-	-	-	-
Trandolapril	3	-	-	-
Trandolapril+candesartan	2	-	-	-
Valsartan	11	-	-	-
Valsartan+amlodipine+canrenone	-	-	-	-
Valsartan+amlodipine+HCTZ	-	-	-	-

*Placebo was ranked 16^th^ in the primary efficacy outcome, 3^rd^ in the cough safety outcome, 2^nd^ in the presyncope safety outcome, and 1^st^ in the peripheral edema safety outcome.

^†^Lisinopril+telmisartan refers to lisinopril+telmisartan combination therapy following lisinopril monotherapy, while telmisartan+lisinopril refers to lisinopril+telmisartan combination therapy following telmisartan monotherapy.

**Fig 3:**
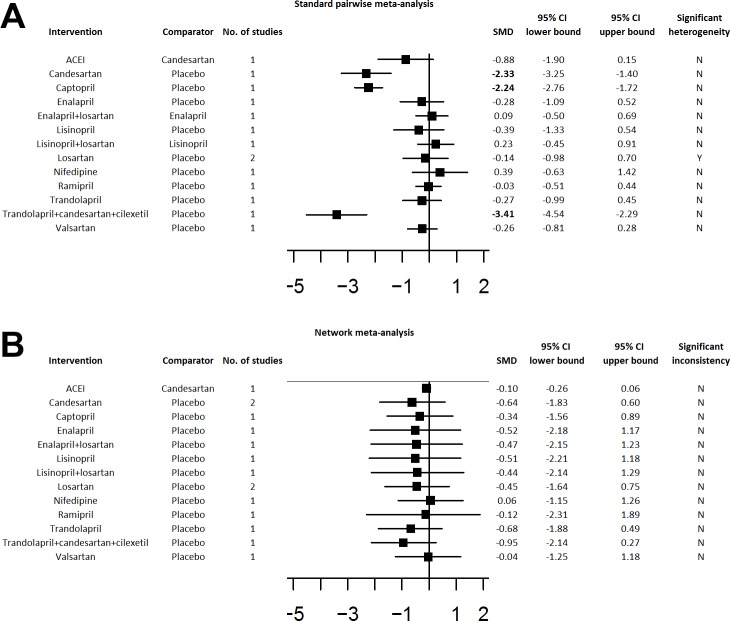
Standard Pairwise and Network Meta-Analyses of Normotensive RCTs. Results from the (A) standard pairwise meta-analysis and (B) network meta-analysis of normotensive RCTs. Standardized mean differences (SMDs) and 95% confidence intervals (CIs) are presented with statistically significant SMDs denoted in bold font. Greyed-out areas represent comparisons that could not be connected into the network. Abbreviations: ACEI, ACE inhibitor (random selection of enalapril, trandolapril, or imidapril); HCTZ, hydrochlorothiazide; TCMZ, trichlormethiazide; SMD, standardized mean difference; 95% CI, 95% confidence interval.

Next, standard pairwise meta-analysis (Fig 3A) and network meta-analysis (Fig 3B) were performed on RCTs with exclusively normotensive participants. The network map diagramming the direct comparisons for this normotensive network meta-analysis is provided in S2 Fig. SUCRA analysis for the primary efficacy outcome of UAER reduction revealed that trandolapril+candesartan was the most efficacious intervention, followed by trandolapril, followed by candesartan (Table 4). SUCRA analysis for the primary safety outcomes revealed that: (i) captopril followed by ramipril were the safest interventions for dry cough; (ii) losartan followed by lisinopril were the safest interventions for presyncope, and (iii) all interventions were equivalently safe to placebo for peripheral edema (Table 4).

**Table 4:** SUCRA-Based Primary Efficacy and Safety Outcome Rankings from Normotensive RCTs.

		Primary safety outcome rankings		
Intervention	Primary efficacy	Dry cough	Presyncope	Peripheral edema
	outcome ranking*	ranking*	ranking*	ranking*
ACEI	-	-	-	-
Candesartan	3	-	-	-
Captopril	9	1	-	-
Enalapril	5	-	-	-
Enalapril+losartan	6	-	-	-
Lisinopril	4	-	-	-
Lisinopril+losartan	8	-	-	-
Losartan	7	-	2	-
Nifedipine	12	-	-	-
Ramipril	13	2	-	-
Trandolapril	2	-	-	-
Trandolapril+candesartan+cilexetil	1	-	-	-
Valsartan	11	-	-	-

*Placebo was ranked 10th in the primary efficacy outcome, was ranked 3rd in the cough safety outcome, was ranked 1st in the presyncope safety outcome, and was not ranked in the peripheral edema safety outcome.

**Fig 4:**
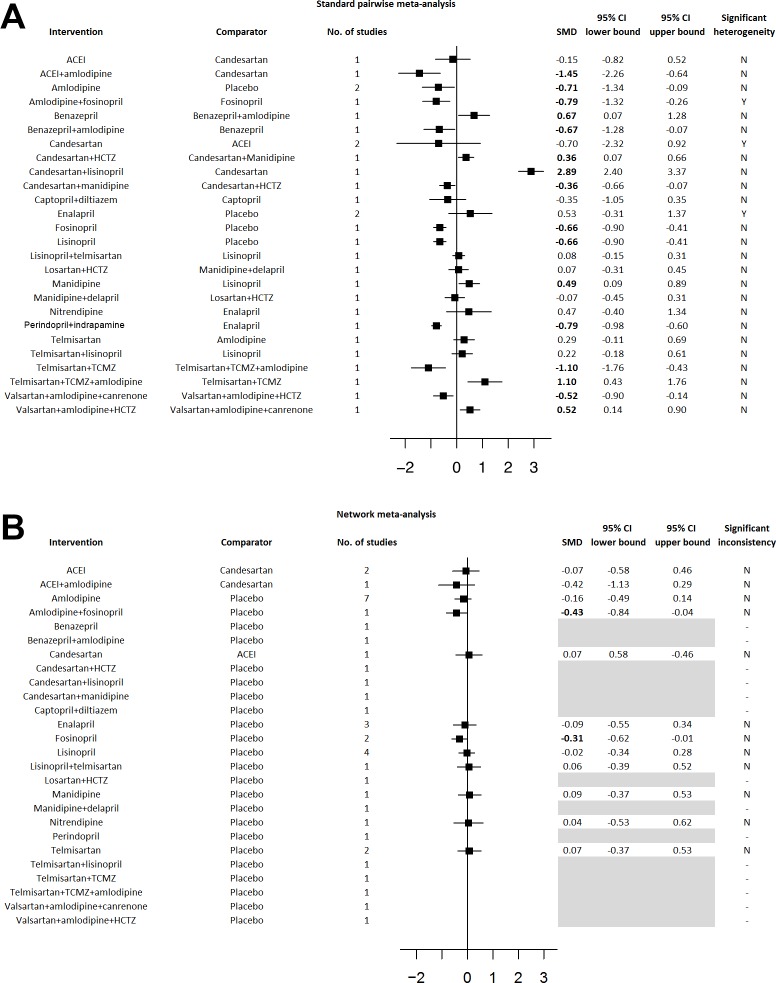
Standard Pairwise and Network Meta-Analyses of Hypertensive RCTs. Results from the (A) standard pairwise meta-analysis and (B) network meta-analysis of hypertensive RCTs. Standardized mean differences (SMDs) and 95% confidence intervals (CIs) are presented with statistically significant SMDs denoted in bold font. Greyed-out areas represent comparisons that could not be connected into the network. Abbreviations: ACEI, ACE inhibitor (random selection of enalapril, trandolapril, or imidapril); HCTZ, hydrochlorothiazide; TCMZ, trichlormethiazide; SMD, standardized mean difference; 95% CI, 95% confidence interval.

Finally, standard pairwise meta-analysis (Fig 4A) and network meta-analysis (Fig 4B) were performed on RCTs with exclusively hypertensive participants. The network map diagramming the direct comparisons for this hypertensive network meta-analysis is provided in S3 Fig. SUCRA analysis for the primary efficacy outcome of UAER reduction revealed that fosinopril+amlodipine was the most efficacious intervention, followed by fosinopril, followed by amlodipine (Table 5). SUCRA analysis for the primary safety outcomes revealed that: (i) amlodipine, followed by fosinopril+amlodipine, followed by fosinopril were the safest interventions for dry cough; (ii) ACEI (random selection of enalapril, trandolapril, or imidapril), followed by lisinopril+candesartan, and followed by candesartan were the safest interventions for presyncope, and (iii) lisinopril+candesartan followed by lisinopril, followed by ACEI (random selection of enalapril, trandolapril, or imidapril) were the safest interventions for peripheral edema (Table 5).

**Table 5:** SUCRA-Based Primary Efficacy and Safety Outcome Rankings from Hypertensive RCTs.

		Primary safety outcome rankings		
Intervention	Primary efficacy	Dry cough	Presyncope	Peripheral edema
	outcome ranking*	ranking*	ranking*	ranking*
ACEI	-	-	1	4
ACEI+amlodipine	-	-	-	-
Amlodipine	3	1	-	-
Amlodipine+fosinopril	1	2	-	-
Benazepril	-	-	-	-
Benazepril+amlodipine	-	-	-	-
Candesartan	-	-	4	5
Candesartan+HCTZ	-	-	-	-
Candesartan+lisinopril	-	-	3	2
Candesartan+manidipine	-	-	-	-
Captopril+diltiazem	-	-	-	-
Enalapril	5	4	-	-
Fosinopril	2	3	-	-
Lisinopril	4	-	5	3
Lisinopril+telmisartan^†^	6	-	-	-
Losartan+HCTZ	-	-	-	-
Manidipine	8	-	-	6
Manidipine+delapril	-	-	-	-
Nitrendipine	10	-	-	-
Perindopril	-	-	-	-
Perindopril+indapamide	-	-	-	-
Telmisartan	9	-	-	-
Telmisartan+lisinopril^†^	-	-	-	-
Telmisartan+TCMZ	-	-	-	-
Telmisartan+TCMZ+amlodipine	-	-	-	-
Valsartan+amlodipine+canrenone	-	-	-	-
Valsartan+amlodipine+HCTZ	-	-	-	-

*Placebo was ranked 7^th^ in the primary efficacy outcome, was not ranked in the cough safety outcome, was ranked 2^nd^ in the presyncope safety outcome, and was ranked 1^st^ in the peripheral edema safety outcome.

^†^Lisinopril+telmisartan refers to lisinopril+telmisartan combination therapy following lisinopril monotherapy, while telmisartan+lisinopril refers to lisinopril+telmisartan combination therapy following telmisartan monotherapy.

Finally, three separate sensitivity analyses were performed to account for any putative effects of type 2 diabetes status, age, or follow-up duration upon the SUCRA analysis for the primary efficacy outcome of UAER reduction. First, the network map diagramming the direct comparisons for the type 2 diabetes sensitivity analysis is provided in S4 Fig. For type 2 diabetes patients with microalbuminuric kidney disease, SUCRA analysis for the primary efficacy outcome revealed that the Tangshen formula was the most efficacious intervention, followed by ramipril, followed by spironolactone (Table 6). Second, the network map diagramming the direct comparisons for the age sensitivity analysis is provided in S5 Fig. For diabetic patients with microalbuminuria aged 40 and over, SUCRA analysis for the primary efficacy outcome revealed that benazepril was the most efficacious intervention, followed by ACEI+amlodipine, followed by candesartan+HCTZ (Table 6). Third, the network map diagramming the direct comparisons for the follow-up duration sensitivity analysis is provided in S6 Fig. For studies with a follow-up duration of 12 months and longer, SUCRA analysis for the primary efficacy outcome revealed that captopril was the most efficacious intervention, followed by perindopril+indapamide, followed by benazepril+amlodipine (Table 6).

**Table 6:** Sensitivity Analyses for the Primary Efficacy Outcome by Diabetes Type, Age, and Follow-Up Duration.

Intervention	Overall SUCRA rank*	SUCRA rank for type 2 diabetes only*	SUCRA rank for aged 40+ only*	SUCRA rank for 12+ months follow-up only*
ACEI	-	7	6	-
ACEI+amlodipine	-	26	2	-
Amlodipine	13	6	-	-
Amlodipine+fosinopril	5	21	-	6
Benazepril	-	20	1	7
Benazepril+amlodipine	-	4	-	3
Candesartan	4	-	7	-
Candesartan+HCTZ	-	15	3	-
Candesartan+lisinopril	-	28	-	-
Candesartan+manidipine	-	5	7	-
Captopril	6	10	4	1
Captopril+diltiazem	1	27	-	14
Enalapril	12	24	4	15
Enalapril+losartan	10	11	-	11
Fosinopril	8	8	2	5
Lisinopril	14	16	-	13
Lisinopril+losartan	15	17	-	8
Lisinopril+telmisartan^†^	18	14	5	-
Losartan	9	18	-	11
Losartan+HCTZ	-	12	8	10
Manidipine	19	23	6	6
Manidipine+delapril	-	25	-	9
Nifedipine	22	12	-	-
Nitrendipine	21	19	-	-
Perindopril	-	15	-	10
Perindopril+indapamide	-	-	-	2
Ramipril	23	2	-	4
Spironolactone	24	3	-	-
Tangshen formula	7	1	-	-
Telmisartan	20	9	3	-
Telmisartan+lisinopril^†^	17	22	-	-
Telmisartan+TCMZ	-	13	-	-
Telmisartan+TCMZ+amlodipine	-	29	-	-
Trandolapril	3	-	-	-
Trandolapril+candesartan	2	-	-	-
Valsartan	11	-	-	12
Valsartan+amlodipine+canrenone	-	-	-	-
Valsartan+amlodipine+HCTZ	-	-	-	-

*Placebo was ranked 16^th^ in the overall analysis, was ranked 30^th^ in the type 2 diabetes analysis, was ranked 9^th^ in the aged 40+ analysis, and was ranked 16^th^ in the 12+ months follow-up analysis.

^†^Lisinopril+telmisartan refers to lisinopril+telmisartan combination therapy following lisinopril monotherapy, while telmisartan+lisinopril refers to lisinopril+telmisartan combination therapy following telmisartan monotherapy.

## Discussion

Here, employing a Bayesian network meta-analytical approach, we assessed the comparative effects of antihypertensive agents in reducing albuminuria in adult diabetic patients with microalbuminuric kidney disease and also specifically examined the comparative effects of antihypertensive agents in reducing albuminuria in normotensive versus hypertensive patients within this microalbuminuric cohort. We found that the ACEI-CCB combination therapy of captopril+diltiazem was the most efficacious intervention for reducing albuminuria irrespective of BP status. However, the ACEI-ARB combination therapy of trandolapril+candesartan was found to be the most efficacious intervention for reducing albuminuria for normotensive patients, while the ACEI-CCB combination therapy of fosinopril+amlodipine was found to be the most efficacious intervention for reducing albuminuria for hypertensive patients. However, the foregoing combination therapies displayed inferior safety profiles relative to ACEI monotherapy with respect to the key adverse side effects of dry cough, presyncope, and peripheral edema.

Palmer et al.’s previous network meta-analysis on adult diabetic patients with CKD identified four classes of antihypertensive agents that are able to significantly regress albuminuria in this patient population (either alone or in combination) [6]: (i) ACEIs (e.g., captopril, trandolapril, fosinopril) [15], (ii) ARBs (e.g., candesartan, losartan) [16], (iii) CCBs (e.g., diltiazem, amlodipine) [17], and (iv) diuretics (e.g., hydrochlorothiazide, trichlormethiazide) [18]. The consensus of clinical evidence has well-established that blockade of the renin-angiotensin-aldosterone system (RAAS) with either an ACEI or ARB agent reduces the risk of adverse renal events in adult diabetic patients with macroalbuminuria (daily albumin excretion of greater than 300 mg) [8]. As several previous clinical trials have found safety issues arising from ACEI-ARB combination therapy including hyperkalemia and acute kidney injury [19–21], the Eighth Joint National Committee (JNC 8) has recommended against ACEI-ARB combination therapy in these patients [22].

Although the majority of the evidence primarily concerns adult diabetic patients with macroalbumuria (daily albumin excretion of greater than 300 mg), there has been some guidance regarding antihypertensive therapy in adult diabetic patients with microalbuminuria (daily albumin excretion of 30–300 mg). The most recent Kidney Disease: Improving Global Outcomes (KDIGO) guidelines for managing diabetic CKD recommends either ACEI or ARB monotherapy for patients with microalbuminuria unless one of the following factors is present: ACEI or ARB therapy is contraindicated, metastatic cancer, treatment of malignancy in the past six months, or admission to a skilled nursing facility in the past three months [23]. The 2014 (Kidney Disease Outcomes Quality Initiative) KDOQI US commentary on these KDIGO guidelines also supports the use of either ACEI or ARB monotherapy based on evidence demonstrating increased harm with ACEI-ARB combination therapy [19, 24].

Interestingly, our current findings reveal that the ACEI-ARB combination therapy of trandolapril+candesartan appears the most efficacious for reducing albuminuria in normotensive adult diabetic patients with microalbuminuria, while the ACEI-CCB combination therapy of fosinopril+amlodipine appears to be the most efficacious intervention for reducing albuminuria for hypertensive adult diabetic patients with microalbuminuria. Therefore, our findings provide an important clarifying distinction to Palmer et al.’s network meta-analysis, which found that ACEI or ARB therapy combined with CCB therapy produces reductions in albuminuria in adult diabetic patients with kidney disease irrespective of BP or microalbuminuric status [6].

Moreover, our safety findings concord with the foregoing KDIGO and KDOQI guidelines regarding combination therapy, as we found that ACEI monotherapy produces a lower likelihood of key adverse outcomes relative to ACEI-ARB or ACEI-CCB combination therapy. For practitioners opting for monotherapy, our SUCRA analysis supports the use of trandolapril in normotensive adult diabetic patients with microalbuminuria and fosinopril in hypertensive adult diabetic patients with microalbuminuria.

With specific respect to type 2 diabetic patients with microalbuminuria, our sensitivity analysis revealed that the Chinese herbal medicine Tangshen formula followed by the ACEI ramipril were the most efficacious interventions for reducing albuminuria. Multiple clinical studies by Ping Li’s research group have demonstrated the efficacy of the Tangshen formula in attenuating diabetic kidney disease in rodent models and human patients [25]. Notably, a 2015 multicenter RCT in type 2 diabetic patients with diabetic kidney disease has also revealed that the Tangshen formula produces a significant decrease in 24-hour urinary protein as well as significant improvements in the estimated glomerular filtration rate (eGFR) relative to placebo [26]. In addition, our finding regarding the efficacy of ramipril ACEI monotherapy in type 2 diabetes patients with microalbuminuria concords with a previous network meta-analysis by Vejakama et al., which demonstrated a superior reno-protective effect with ACEI monotherapy or ARB monotherapy over CCB monotherapy or placebo in type 2 diabetic patients [27].

There are several limitations to this study. First, the three separate sensitivity analyses on type 2 diabetes status, age, and follow-up duration revealed that each of these three factors had a significant impact upon the findings. That being said, across all sensitivity analyses, ACEI monotherapy was found to be superior to all other antihypertensive medication classes (with the notable exception of the Tangshen formula for type 2 diabetes patients with microalbuminuria). Second, we could not assess the degree of albuminuria reduction attributable to a medication class effect beyond BP reduction. We considered including target/achieved BP as part of our analysis; however, the included RCTs did not report this data to a sufficient degree to enable such an analysis in the current study. Third, as the response to different medication classes may vary with excretory renal function, a subgroup analysis according to the presence or absence of reduced excretory renal function would provide valuable insights. Unfortunately, this analysis was not possible with the available data. Fourth, although we were able to assess the effects of various hypertensive agents upon regressing UAER, the available reported data in the included studies did not enable us to estimate the effects of antihypertensive agents on other clinically relevant renal outcomes. For example, we were unable to examine acute kidney injury as a safety endpoint due to the paucity of reported data on this outcome. Fifth, as most of the included RCTs were from developed Western countries and Japan, there is a scarcity of data from other countries, which may have biased our conclusions. Finally, drug dosing was not controlled for in the meta-analyses; most of the included RCTs allowed the clinical investigators to titrate drug dosing for participants.

In conclusion, the ACEI-ARB combination therapy of trandolapril+candesartan appears the most efficacious for reducing albuminuria in normotensive diabetic patients with microalbuminuria, while the ACEI-CCB combination therapy of fosinopril+amlodipine appears to be the most efficacious intervention for reducing albuminuria for hypertensive diabetic patients with microalbuminuria. However, the foregoing combination therapies displayed inferior safety profiles to ACEI monotherapy with respect to the key adverse side effects of dry cough, presyncope, and peripheral edema. For practitioners opting for monotherapy, our SUCRA analysis supports the use of trandolapril in normotensive adult diabetic patients with microalbuminuria and fosinopril in hypertensive adult diabetic patients with microalbuminuria.

## Supporting Information

S1 Fig Network Map of the Overall Network Meta-Analysis. Networked interventions are placed in green boxes, while non-networked interventions are placed in grey boxes. Blue lines between interventions indicate direct comparisons with the number of studies indicated.
(TIF)

S2 Fig Network Map of the Normotensive Network Meta-Analysis. Networked interventions are placed in green boxes, while non-networked interventions are placed in grey boxes. Blue lines between interventions indicate direct comparisons with the number of studies indicated.
(TIF)

S3 Fig Network Map of the Hypertensive Network Meta-Analysis. Networked interventions are placed in green boxes, while non-networked interventions are placed in grey boxes. Blue lines between interventions indicate direct comparisons with the number of studies indicated.
(TIF)

S4 Fig Network Map of the Type 2 Diabetes Sensitivity Analysis. Networked interventions are placed in blue circles with the circle size reflecting the relative study size. Black lines between interventions indicate direct comparisons with thicker lines indicating a larger number of comparator studies.
(TIF)

S5 Fig Network Map of the Age Sensitivity Analysis. Networked interventions are placed in blue circles with the circle size reflecting the relative study size. Black lines between interventions indicate direct comparisons with thicker lines indicating a larger number of comparator studies.
(TIF)

S6 Fig Network Map of the Follow-Up Duration Sensitivity Analysis. Networked interventions are placed in blue circles with the circle size reflecting the relative study size. Black lines between interventions indicate direct comparisons with thicker lines indicating a larger number of comparator studies.
(TIF)
