# Supplementary figures and images for: Comparative Efficacy and Safety of Antihypertensive Agents for Adult Diabetic Patients with Microalbuminuric Kidney Disease: A Network Meta-Analysis
Source: PLoS One. 2017 Jan 3;12(1):e0168582. doi: 10.1371/journal.pone.0168582 (PMC5207630; doi:10.1371/journal.pone.0168582)

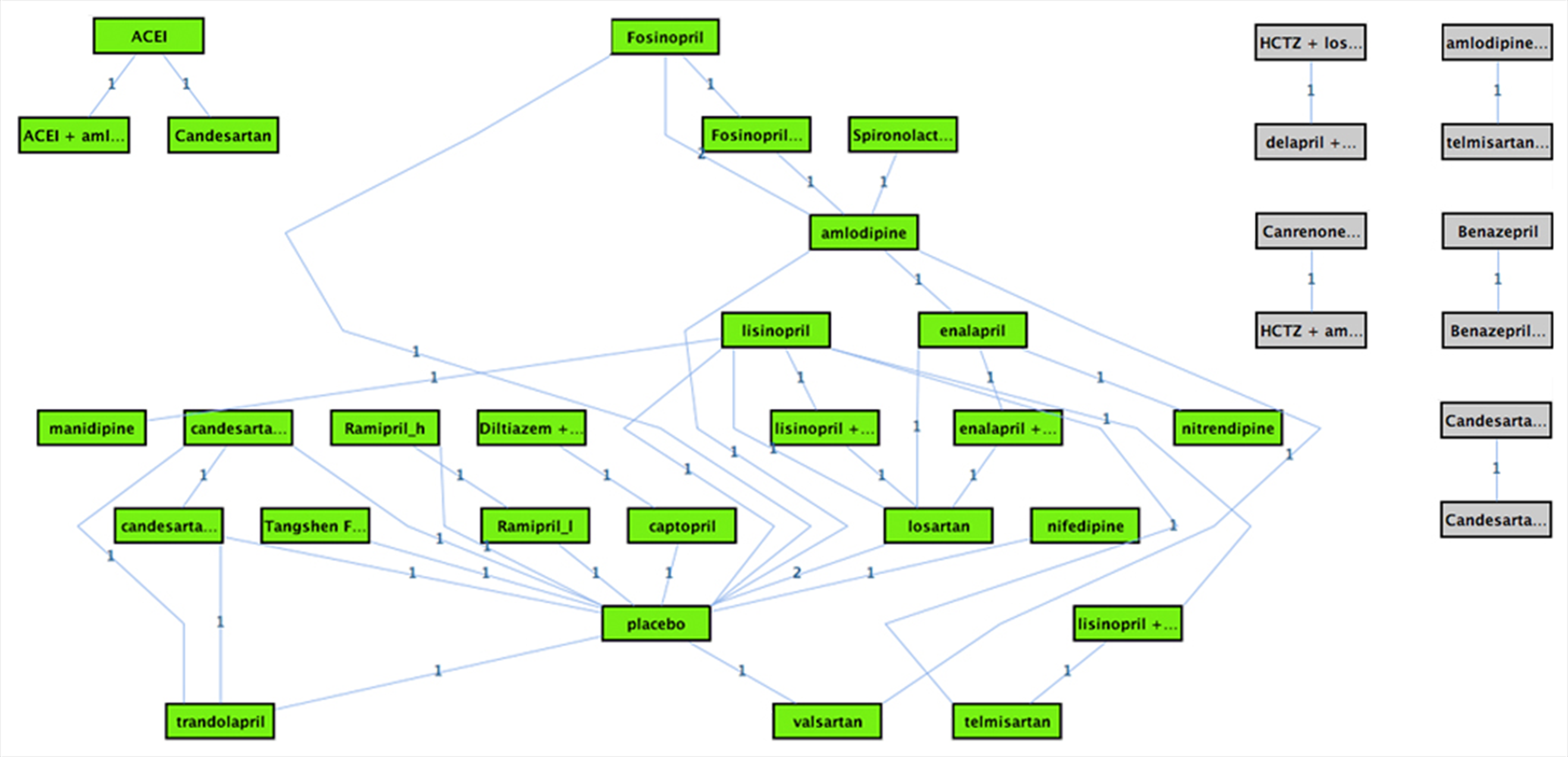

Supplement: S1 Fig — Networked interventions are placed in green boxes, while non-networked interventions are placed in grey boxes. Blue lines between interventions indicate direct comparisons with the number of studies indicated. (TIF) [file pone.0168582.s001.tif]

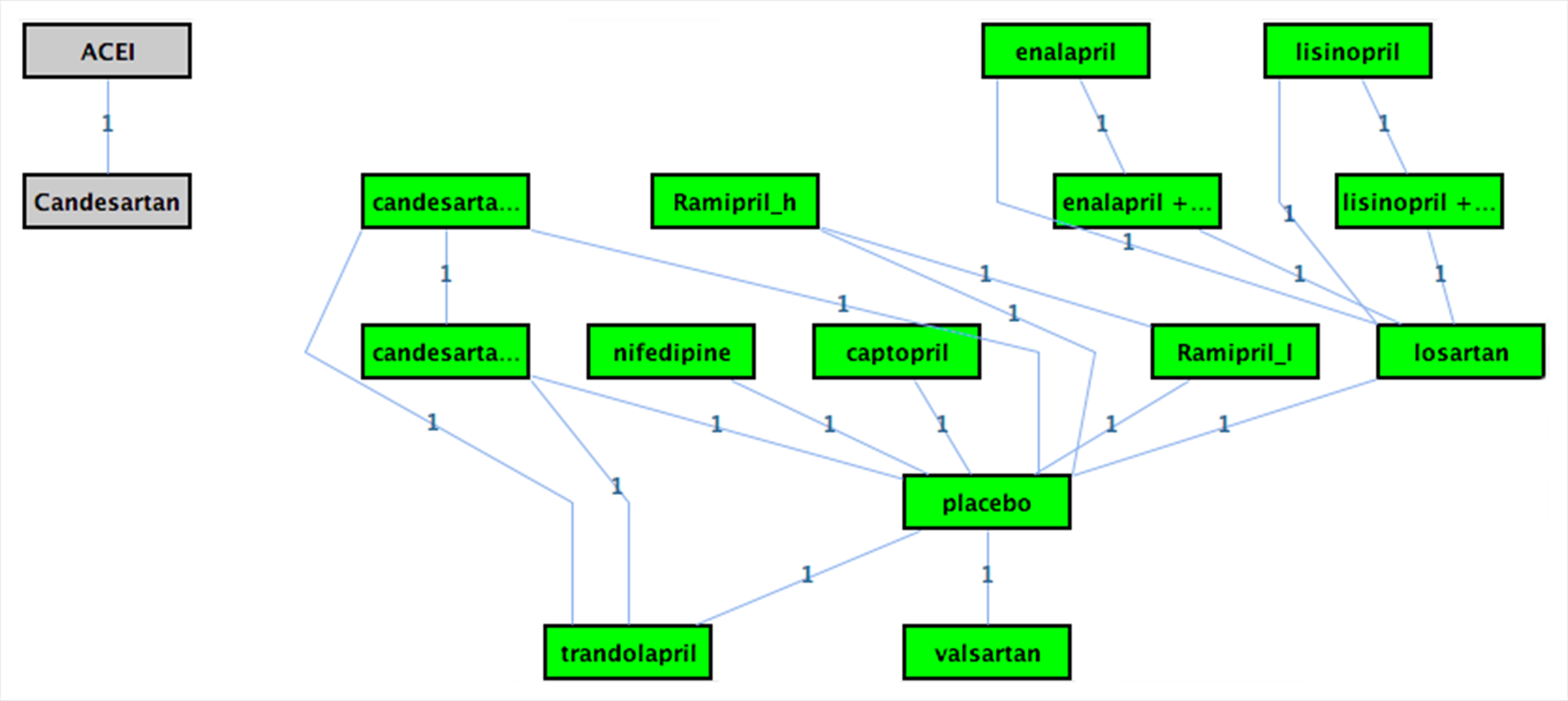

Supplement: S2 Fig — Networked interventions are placed in green boxes, while non-networked interventions are placed in grey boxes. Blue lines between interventions indicate direct comparisons with the number of studies indicated. (TIF) [file pone.0168582.s002.tif]

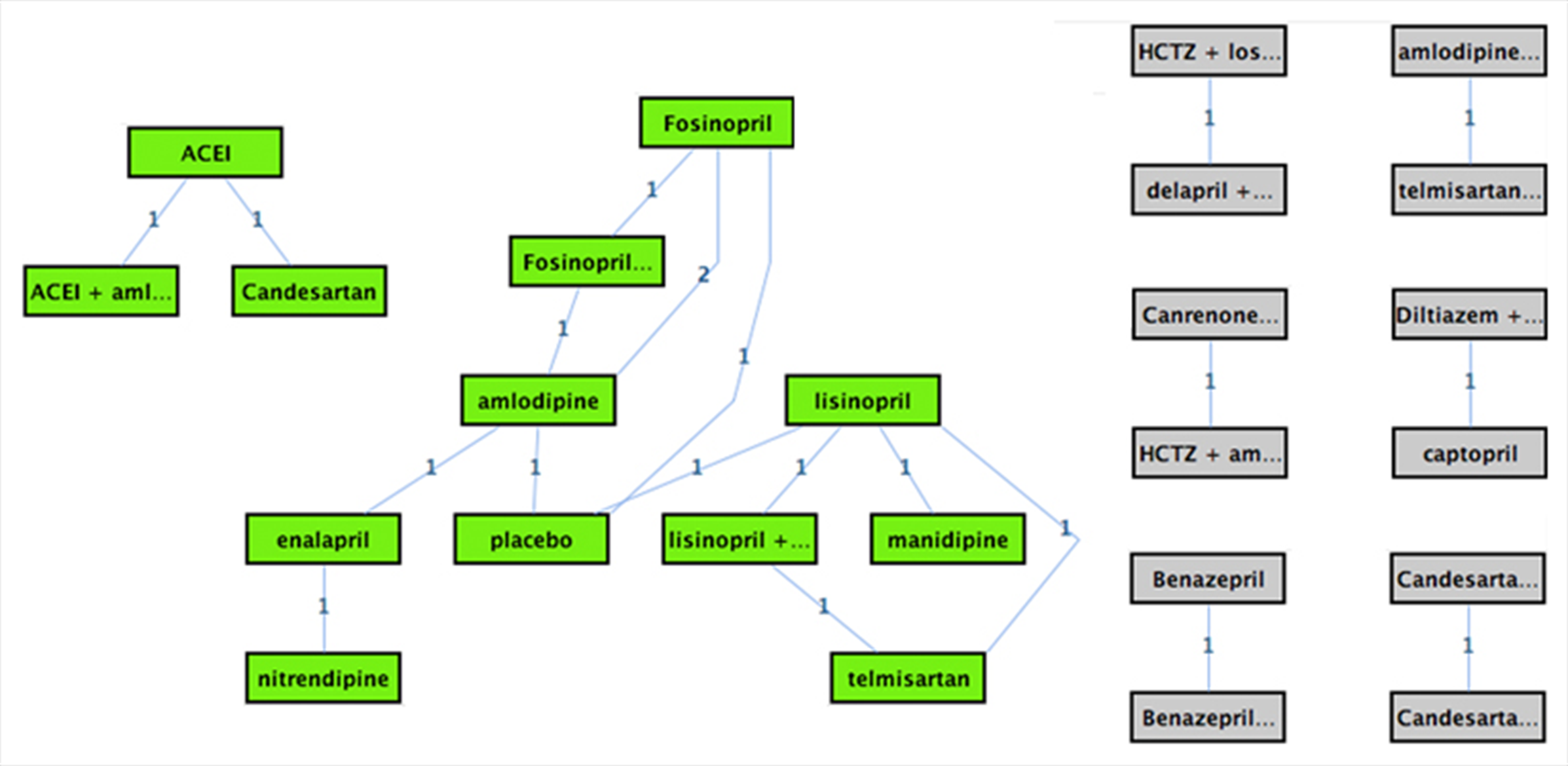

Supplement: S3 Fig — Networked interventions are placed in green boxes, while non-networked interventions are placed in grey boxes. Blue lines between interventions indicate direct comparisons with the number of studies indicated. (TIF) [file pone.0168582.s003.tif]

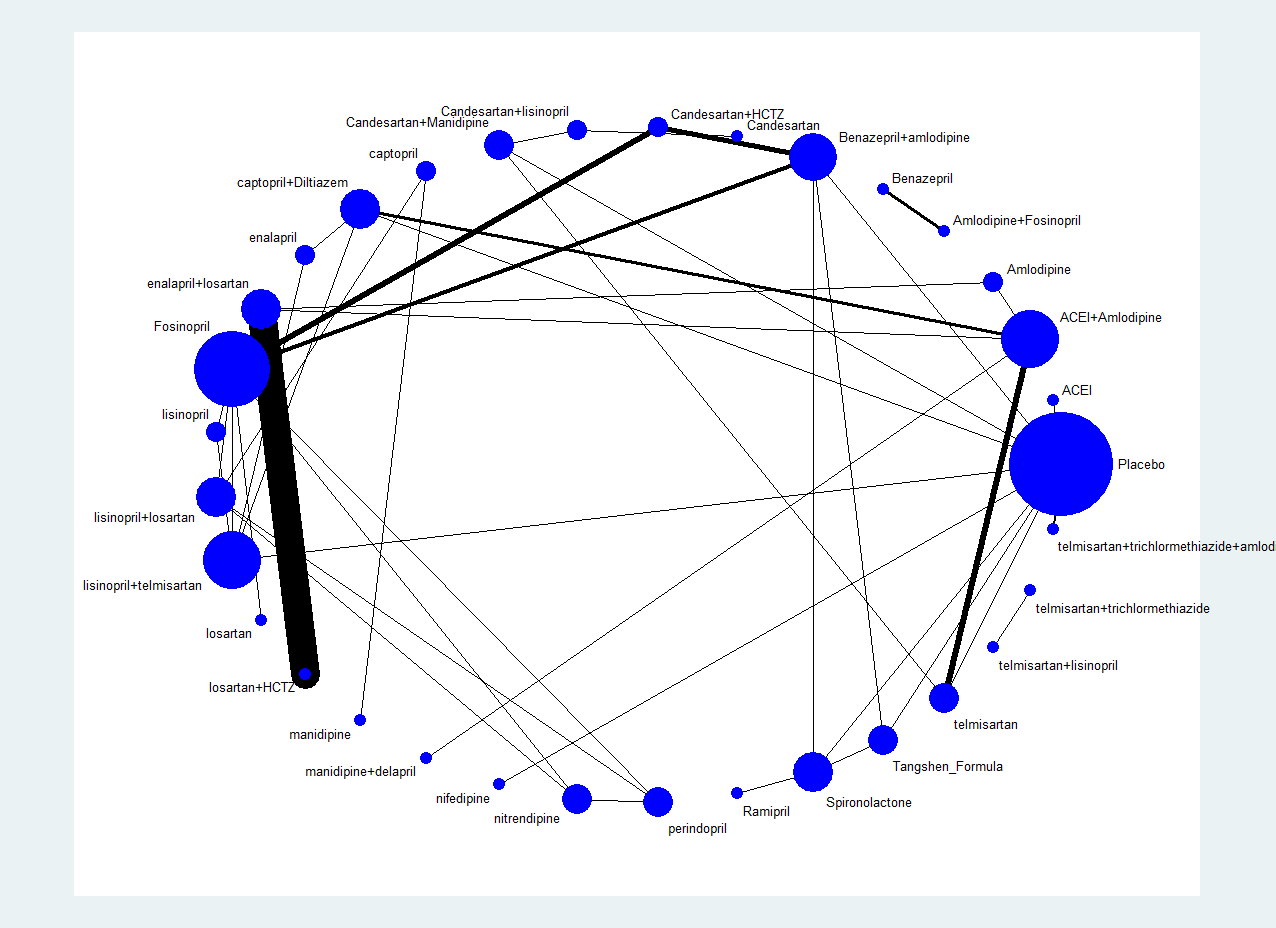

Supplement: S4 Fig — Networked interventions are placed in blue circles with the circle size reflecting the relative study size. Black lines between interventions indicate direct comparisons with thicker lines indicating a larger number of comparator studies. (TIF) [file pone.0168582.s004.tif]

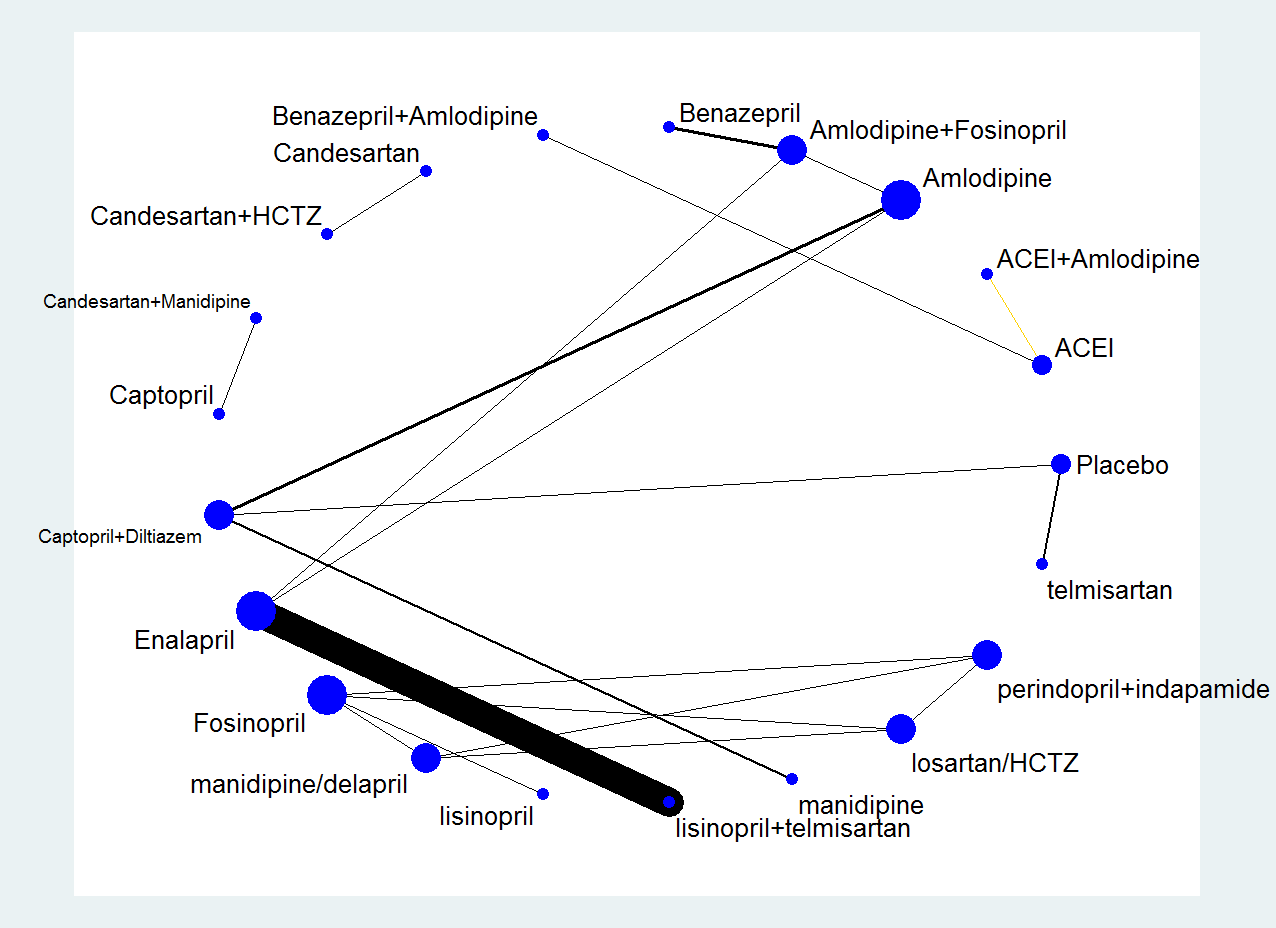

Supplement: S5 Fig — Networked interventions are placed in blue circles with the circle size reflecting the relative study size. Black lines between interventions indicate direct comparisons with thicker lines indicating a larger number of comparator studies. (TIF) [file pone.0168582.s005.tif]

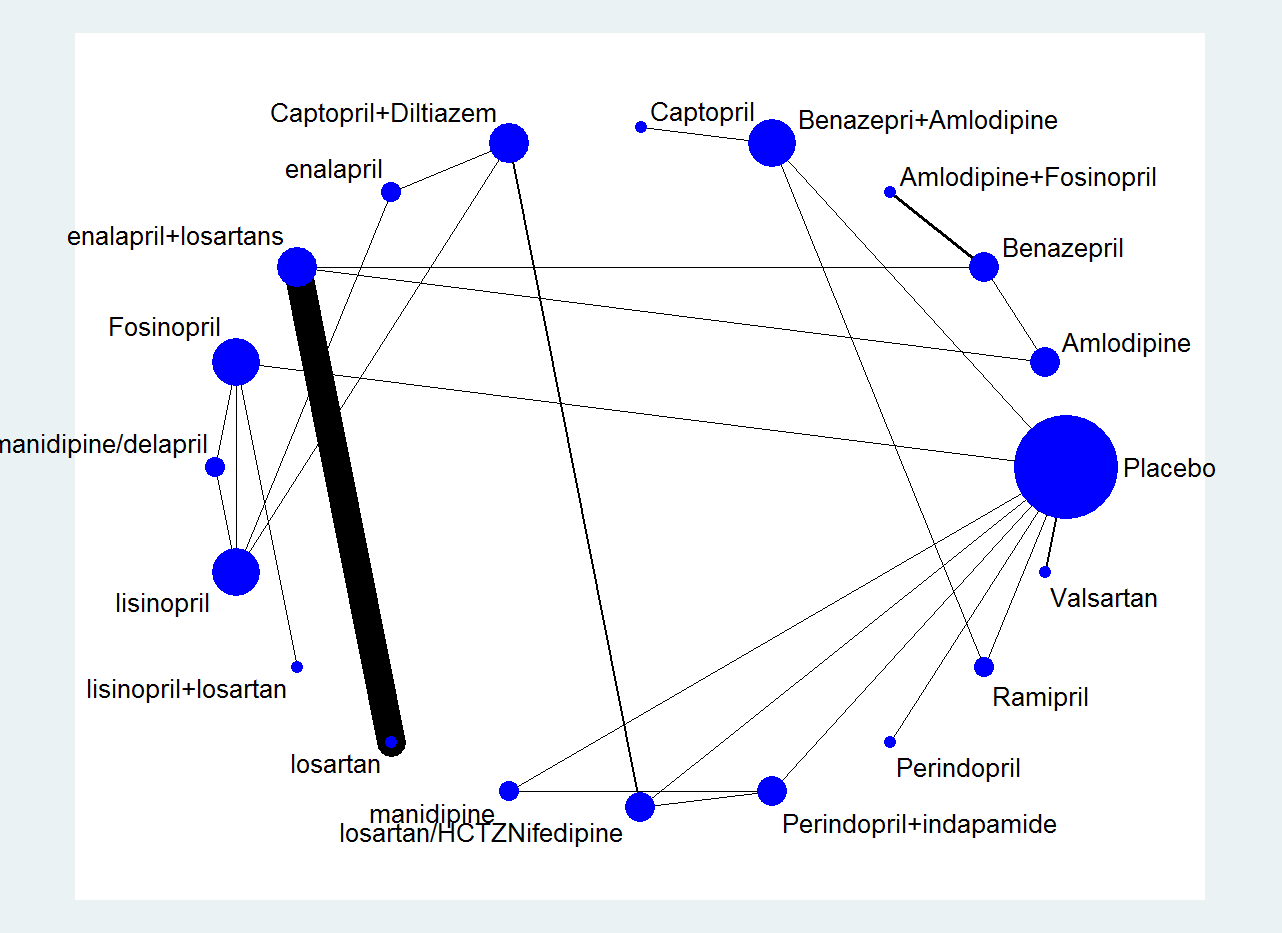

Supplement: S6 Fig — Networked interventions are placed in blue circles with the circle size reflecting the relative study size. Black lines between interventions indicate direct comparisons with thicker lines indicating a larger number of comparator studies. (TIF) [file pone.0168582.s006.tif]
